# Supplementary material for: Optical-Tweezers-integrating-Differential-Dynamic-Microscopy maps the spatiotemporal propagation of nonlinear strains in polymer blends and composites
Source: Nat Commun. 2022 Sep 2;13:5180. doi: 10.1038/s41467-022-32876-y (PMC9440072; doi:10.1038/s41467-022-32876-y)
Supplement: Supplementary file 1 — Supplementary Information [file 41467_2022_32876_MOESM1_ESM.pdf]

OpTiDDM maps the spatiotemporal propagation of nonlinear strains in polymer blends and composites

Karthik R. Peddireddy, Ryan Clairmont, Philip Neill, Ryan McGorty, and Rae M. Robertson-Anderson*

Department of Physics and Biophysics, University of San Diego, 5998 Alcala Park, San Diego, CA 92110, United States

Supplemental Information

Section S1. Expanded Methods Section

Section S2. Predicted length and time scales

Figure S1. Confocal micrographs of 0.5:6.4 DNA-MT composite.

Figure S2. Relaxation timescales for the DNA blends (0.65:0, 0.5:0) and DNA-MT composite (0.5:6.4), determined by fitting force relaxation curves to a sum of exponentials.

Figure S3. Instantaneous alignment factor values over the course of the entire strain program comprising 6-9 periods of strain and cessation.

Figure S4. OpTiDDM measurements in water.

Section S1. Expanded Methods Section

DNA: Double-stranded, 115 kilobasepair (kbp) DNA, is prepared by replication of cloned bacterial artificial chromosomes (BACs) in *Escherichia coli*, followed by extraction, purification, concentration and resuspension in TE10 buffer (10 mM Tris-HCl (pH 8), 1 mM EDTA, 10 mM NaCl) using custom-designed and validated protocols[1,2]. We use gel electrophoresis image analysis to quantify the mass concentration of the resulting DNA stock solutions c_D and the corresponding mass fractions f of relaxed circular (ring, R) and linear (L) topologies (no supercoiled constructs are present). Using Life Technologies E-Gel Imager and E-Gel GelQuant Express Analysis software we determine $c \simeq 0.8$ mg/ml, $f_L \simeq 0.5$ and $f_R \simeq 0.5$. We perform no enzymatic digestion after this point as we aimed to study ring-linear blends with $f_L \simeq f_R$ due to the intriguing emergent mechanical properties that have been previously reported in ring-linear blends of comparable fractions[3-6]. For experiments, we use two different dilutions of this stock: $c_{D,\downarrow} = 0.5$ mg/ml and $c_{D,\uparrow} = 0.65$ mg/ml.

The contour lengths of the ring and linear DNA are fixed at $L =$115 kbp (~39 μm), but due to their end-closure, rings have a ~$1.6x$ smaller radius of gyration R_G than their linear counterparts[1]. Using the previously reported values of $R_{G,R} \simeq 541$ nm and $R_{G,L} \simeq 885$ nm for ring and linear 115 kbp DNA[1], we determine the polymer overlap concentration of the ring-linear DNA blend via $c_{RL}^* = (3/4\pi)(M/N_A)/(f_L R_{G,L}^3 + f_R R_{G,R}^3)$ where M is the DNA molecular weight[7]. Our computed value of $c_{RL}^* \simeq 710$ μg/mL yields $c_{D,\downarrow} \simeq 7c_{RL}^*$ and $c_{D,\uparrow} \simeq 9c_{RL}^*$ for the two DNA concentrations we use in experiments.

To image DNA for DDM, we fluorescent-label a small fraction of the DNA molecules with MFP488 (Mirus) using the manufacturer-supplied *Label* IT Labeling Kit and corresponding protocols (Mirus). The excitation/emission spectrum for MFP488 is 501/523 nm and the dye molecule to DNA base pair ratio is 5:1.

Microtubules (MT): Unlabeled porcine brain tubulin (T240) and rhodamine-labeled porcine brain tubulin (TL590M) are obtained from Cytoskeleton, Inc. 45.5 μM stock solutions of tubulin dimers, containing 9:1 unlabeled:labeled tubulin dimers in PEM100 buffer (100 mM PIPES (pH 6.8), 2 mM $MgCl_2$, 2mM EGTA) are flash-frozen in and stored at -80°C. To form the DNA-MT composite, we add a molar concentration of $c_T = 6.4$ μM tubulin dimers to the 0.5 mg/ml DNA solution, followed by 2 mM GTP and 10 μM Taxol to polymerize the tubulin into microtubules and stabilize the MTs[8]. The formed MTs are hollow rods with a diameter $D \simeq 25$ nm comprising 13 tubulin dimers per ring[9].

Sample preparation: We perform measurements on three different systems, which we denote by the ratio of the DNA mass concentration (in mg/ml) and tubulin molar concentration (in μM): c_D: $c_T =$ 0.5:0, 0.65:0, and 0.5:6.4. We add 10 μg/ml MFP488-labeled DNA and a trace amount of polystyrene beads with radius $b =$2.25 μm (Polysciences, Inc.) to enable DDM and OpT measurements, respectively. The concentration of labeled DNA is optimized for DDM measurements as described below and depicted in Fig 1c. Beads are coated with AlexaFluor594-BSA (ThermoFisher) to prevent DNA adsorption and enable fluorescence imaging. Note that beads and DNA are labeled with distinct excitation/emission dyes such that the beads do not interfere with DDM measurements of DNA tracers, and the fluorescence lifetimes of the DNA tracers are prolonged while moving to a new bead and location in the chamber. An oxygen scavenging system (45 μg/mL glucose, 43 μg/mL glucose oxidase, 7 μg/mL catalase, and 5 μg/mL β-mercaptoethanol) is added to inhibit photobleaching. Sample chambers (20×3×0.1 mm^3) are made with a microscope glass slide and coverslip, each coated with BSA to prevent adsorption of DNA, MTs and beads, and separated by two layers of double-sided tape. All samples are mixed slowly and thoroughly, using wide-bore pipette tips to prevent shearing of DNA, then introduced into sample chambers through capillary action and hermetically sealed with epoxy. For the DNA-MT composite (c_D: $c_T =$ 0.5:6.4), tubulin dimers are added immediately before flowing into the chamber, and the sample chamber is incubated at 37°C for 2 hours, resulting in repeatable and reliable tubulin polymerization in the DNA solution (see SI Fig S1)[8].

OpTiDDM instrumentation and protocol: We use a custom-built optical trap formed from a 1064 nm Nd:YAG fiber laser (Manlight) focused with a 60× 1.4 NA objective (Olympus) and integrated into an Olympus IX71 epifluorescence microscope[10]. The force exerted by the sample on a trapped bead is determined by measuring the laser beam deflections via a position sensing detector (PSD, First Sensor) at 20 kHz. The trap is calibrated for force measurement using the Stokes drag method[11]. To image the MPF488-labeled DNA and AlexaFluor594-labeled microspheres in the samples we use 490/525 nm and 530/575 nm excitation/emission filter cubes and an ORCA-Flash 4.0 LT+ CMOS camera (Hamamatsu). For DDM measurements, we use a piezoelectric actuator mirror (PI USA) to move the trap relative to the sample chamber while keeping the 600×900 square-pixel (130 nm/pixel) field-of-view (FOV) of the camera fixed and centered at the resting trap position. For force measurements, performed independently of DDM measurements, we use a piezoelectric nanopositioning stage (Mad City Laboratories) to move the sample relative to the fixed trap. All device control and data acquisition is performed using National Instruments Labview 2019 version 19.0.1f1 (64 bit).

As shown in Figure 1, the microrheological strain program we apply consists of repeatedly moving the trapped bead back and forth horizontally (along the x-axis) through a strain distance $s = 15$ µm at roughly logarithmically spaced strain rates of $\dot{\gamma} = 9.4$, 19, 42, 90, 189 s^{-1} which correlate to speeds of v = 10 – 200 µm/s via the relation $\dot{\gamma} = 3v/\sqrt{2}b$ (9.4-189 s^{-1})[12]. We pause between each 15 µm sweep for a fixed cessation time Δt_R = 3 s to allow the polymers to relax. Specifically, we set Δt_R to be comparable to the longest measurable relaxation time of the systems (SI Fig S2). Below this timescale the system would still be relaxing at the onset of the next strain in the cycle, complicating the resulting dynamics, while longer cessation times would unnecessarily prolong measurements, thereby reducing the number of cycles possible before the photobleaching becomes prohibitive. We determine this relaxation time by performing single sweep measurements with 60 s cessation periods and fitting the force relaxation curves to a sum of exponentials, as described in SI Fig S2[3,8,13,14].

We perform each oscillatory strain program for a total time of 50 s, and do not start a new sweep unless there is enough time to complete a full forward-backward cycle in the allotted 50 s time period. These criteria result in 6,7,8,8 and 9 complete cycles for the $\dot{\gamma} = 9.4$, 19, 42, 90, and 189 s^{-1} strains, respectively. For each measurement ≥10 trials are conducted, each with a new microsphere in a new unperturbed location. Presented data is an average of all trials. During each 50 s strain, we capture a time-series of 78 µm ×117 µm images (centered on the strain path) of the labeled DNA in the sample at 60 fps, and record the PSD signal (i.e., laser deflections) at 20 kHz.

We use custom-written Matlab R2021a scripts, available at https://github.com/kpeddire/OptiDDM.git to process and analyze the acquired force data we present in Figures 6-8, S2 and S4.

Differential Dynamic Microscopy (DDM): To determine how the strain-induced dynamics and structure of the polymeric fluids depend on the orthogonal distance y from the strain path (Figs 1-5), we divide the FOV into 128×128 square-pixel (16.6 µm)^2 ROIs, centered horizontally at the midpoint of the $s = 15$ µm strain path and shifted along the $\pm y$ direction in 16-pixel increments, with the bottom edge of the first ROI at $y = 0$ (and its center at $y = 8$ µm) and the farthest ROI centered at $y = 28$ µm. We analyze 10 ROIs in each of the $+y$ and $-y$ directions and average the $+/-$ data for each y, as they exhibit statistically indistinguishable dynamics (as we expect).

We note that our use of wide-field fluorescence microscopy for imaging has the limitation that signal from out-of-focus planes can contribute to the images. We optimize the density of DNA labels such that they are dense enough for ample statistics in the small ROIs necessary to achieve sufficient spatial resolution, while dilute enough such that polymer fluctuations are resolvable and the imaging is not overwhelmed by signal from out-of-focus planes. Fig 1c shows an example of an optimized DNA labeling density. Nevertheless,

should one require increased signal-to-noise, OpT could be coupled with confocal or light-sheet microscopy that reduce out-of-focus signal[15-18]. These modalities may also prove useful for visualizing strain-induced polymer clustering and spatial heterogeneities that we do not characterize here.

We use custom-written Python2.7 scripts, available at https://github.com/kpeddire/OptiDDM.git, to perform DDM analysis[19]. These scripts take two-dimensional Fourier transforms of differences between images separated by a range of lag times Δt to quantify how the degree of correlation decays with Δt as a function of the wavevector q, which we quantify via a 3D image structure function $D(\mathbf{q}, \Delta t)$.

To determine the extent to which the DNA dynamics are preferentially aligned along the strain path (x-axis) (Fig 2), we adopt alignment factor analysis typically used to analyze scattering data produced by an aligned field[20,21]. We compute an alignment factor A_F with respect to the strain path (x-axis) by computing weighted azimuthal integrals of the 2D image structure function $D(q_x, q_y, \Delta t)$ (i.e., integrals over θ where $\theta = \tan^{-1}(q_y/q_x)$) where q_x and q_y are x and y components of the wavevector $\mathbf{q}$: $A_F(q, \Delta t) = \int_0^{2\pi} D(q, \Delta t, \theta) \cos(2\theta)\, d\theta \Big/ \int_0^{2\pi} D(q, \Delta t, \theta) d\theta$ (Fig 1f). Here, θ is defined relative to the x-axis such that isotropic and completely x-aligned dynamics correspond to $A_F = 0$ and $A_F = A(q)/(A(q) + 2B(q))$, respectively, where $A(q)$ and $B(q)$ are amplitude and background terms described below. Increasing A_F values indicate more alignment. The ratio A/B is a measure of the signal-to-noise of the system, such that when this ratio is high (i.e., $A \gg B$), $A_F \to 1$ for complete x-alignment. For our data, $A/B \lesssim 2$ such that $A_F \lesssim 0.5$.

To obtain a single A_F value for each distance y, we average over Δt = 0.17-1 s and q =1-7 μm^{-1}, where there is no statistically significant dependence of A_F on these parameters. We note that the maximum A_F values we measure are ~0.02, significantly lower than the theoretical maximum of ~0.5 for our setup. One potential source of these low values is our choice of ROI sizes of (16 μm)^2, which we optimized to ensure ample statistics and signal-to-noise, but which are significantly larger than the intrinsic lengthscales of the systems. Even our measurement closest to the strain includes molecules that are ~10 tube diameters ($d_T \approx$ 1.6 μm) from the strain, so likely are much less aligned than those ~d_T from the strain. Time-averaging adds an additional contribution to potentially lowering A_F as described below and in SI Fig S3.

To determine the type and rate of motion of the DNA, we radially average each $D(\mathbf{q}, \Delta t)$ to get a 1D image structure function that can be described by $D(q, \Delta t) = A(q)[1 - f(q, \Delta t)] + B(q)$, where $f(q, \Delta t)$ is the intermediate scattering function (ISF), $A(q)$ is the amplitude, and $B(q)$ is the background. While radial averaging is only strictly valid for isotropic $D(\mathbf{q}, \Delta t)$ functions, which is admittedly a crude approximation in cases in which A_F is large, we note that the largest A_F value we measure is ~0.02, so the anisotropy is relatively weak even in the most extreme cases (see SI Fig S3).

To determine the type and rate of motion, we model the ISF as a stretched exponential: $f(q, \Delta t) = e^{-(\Delta t/\tau(q))^{\delta}}$ where $\tau(q)$ is the decay time and δ the stretching exponent. Stretched (δ >1) and compressed (δ <1) exponentials, as opposed to simple exponentials (δ =1), have been shown to describe crowded, entangled[22-26] and active systems[27-29], respectively. By evaluating the functional form of $\tau(q)$ determined from fitting the ISF, we analyze the extent to which $\tau(q)$ can be described by power-law scaling $\tau(q) \sim q^{-\beta}$ where the scaling exponent β describes the type of motion. Specifically, $\beta = 2$ is indicative of normal Brownian diffusion whereas $\beta \to 1$ describes superdiffusive or ballistic dynamics and $\beta > 2$ indicates anomalous subdiffusion[30,31].

We note that for all DDM analyses, we average over the entire time of the cyclic strain, which includes both strain and cessation periods. While one could evaluate the strain and cessation periods separately, the analysis is much more laborious and less user-friendly. We choose to not complicate the analysis in this

way to facilitate other researchers in adopting our approach. Moreover, we expect the trends we measure to be largely insensitive to our averaging approach, as indicated by the statistically significant trends we present as well as the time-dependent A_F curves shown in SI Fig S3.

Section S2. Predicted length and time scales

According to traditional reptation theory, the tube radius for linear polymers is computed using the relation $a_L = (24N_e/5)^{1/2}R_{G,L}$, where the number of entanglements per chain N_e is given by $N_{e,L} = 4cRT/5MG_N^0$ where G_N^0 is the elastic plateau modulus[7]. Using our previously reported value of $G_N^0 \simeq$0.2 Pa for linear 115 kbp DNA at $c = 1$ mg/ml[32], and the accepted scaling $G_N^0 \sim c^2$ for entangled linear polymers[7], we estimate a_L for the $f_L \simeq$ 0.5 fraction of linear DNA in the 0.5:0 and 0.65:0 blends (i.e., ~0.25 mg/ml and ~0.375 mg/ml) as $a_L \simeq$ 1.4 µm and ~1.2 µm, respectively. To estimate the corresponding tube radius for the $f_R \simeq$ 0.5 ring fraction, we use the pom-pom ring model prediction $a_R/a_L = (5N^{-0.4})^{1/2}$, where $N =$ 382 is the number of Kuhn lengths[33], to compute $a_R \simeq$ 0.95 µm and ~0.84 µm for $c_D = 0.5$ mg/ml and 0.65 mg/ml, respectively. Finally, to estimate an effective tube diameter $d_T = 2a$ for each $f_L \simeq f_R \simeq 0.5$ blend, we use the relation $d_T^{-3} = [(2a_L)^{-3} + (2a_R)^{-3}]$ that considers the density of each cubic tube diameter ${d_T}^3$ to arrive at values of $d_T \simeq$ 1.74 µm and ~1.53 µm for the 0.5:0 and 0.65:0 blends, respectively[14]. For reference, if we assume that the blends are comprised entirely of linear chains we compute tube diameters of $d_{T,L} \simeq$ 1.98 µm and ~1.73 µm for the 0.5:0 and 0.65:0 blends, respectively.

We can approximate the theoretical entanglement time τ_e, or the time it takes for a linear entangled polymer to 'feel' its entanglement tube, based on the predicted expression for entangled linear polymers: $\tau_e \simeq (N_e R_G/D_0)^2$ where D_0 is the dilute limit diffusion coefficient[7], providing $\tau_{e,0.5} \simeq$ 15.7 ms and $\tau_{e,0.65} \simeq$ 26.6 ms for the 0.5:0 and 0.65:0 blends, respectively. Within the same tube theory framework, we can estimate the disengagement time τ_D, i.e. the longest predicted relaxation time for entangled linear polymers, using $\tau_D \simeq (36R_G^4/\pi^2 a^2 D_0)$, providing $\tau_{D,0.5} \simeq$ 18.5 s and $\tau_{D,0.65} \simeq$ 24.2 s [7].

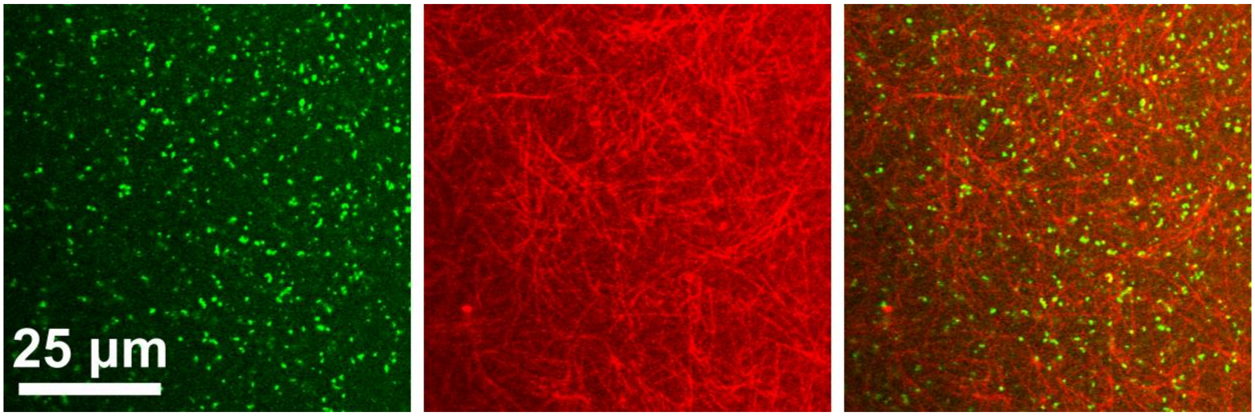

Figure S1. Confocal micrographs of 0.5:6.4 DNA-MT composite. Confocal micrographs (71 µm × 71 µm) of a DNA-MT composite with 6.4 µM rhodamine-labeled tubulin polymerized into microtubules in a solution of 0.5 mg/ml DNA (comprising a 1:1 ratio of ring and linear topologies). The green (left) and red (middle) images show MFP488-labeled DNA and rhodamine-labeled microtubules in the 0.5:6.4 composite, and the rightmost figure is the composite of the two channels. The scale bar applies to all images. The 512×512 square-pixel images (1 pixel = 0.14 µm) are acquired using a Nikon A1R laser scanning confocal microscope with a 60× 1.4 NA objective. 10% of tubulin dimers comprising microtubules are rhodamine-labeled to enable imaging using a 561 nm laser with 561 nm excitation and 595 nm emission filters. MFP488-labeled DNA, comprising 10% of the total DNA in solution, are imaged using a 488 nm laser with 488 nm excitation and 525 nm emission filters. Each micrograph is representative of reproducible data collected for >5 independent samples.

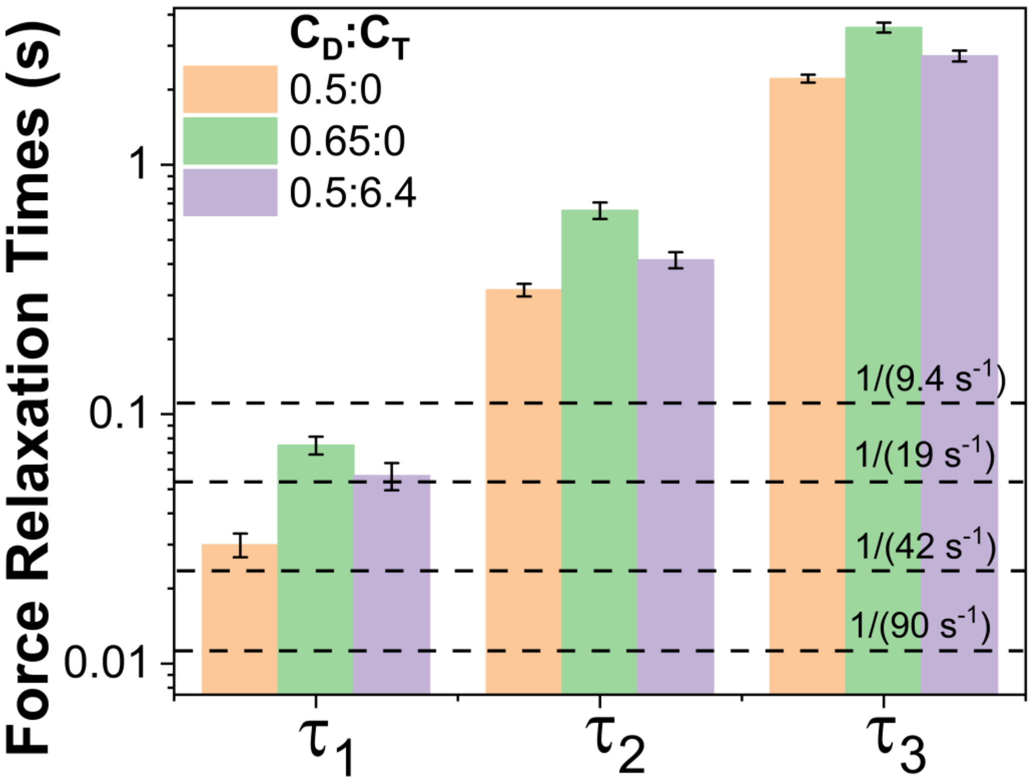

Figure S2. Relaxation timescales for the DNA blends (0.65:0, 0.5:0) and DNA-MT composite (0.5:6.4), determined by fitting force relaxation curves to a sum of exponentials. Following the approach detailed in SI Refs 1-4, we modify our cyclic strain program to allow the system to relax for $\Delta t_R = 60$ s after the applied strain to capture the full spectrum of relaxation times. Measurements are performed at 4 strain rates, the inverse of which are indicated by the dashed horizontal lines. We fit each force relaxation curve to a sum of three exponential decays and a residual: $F(t) = F_\infty + C_1 e^{-t/\tau_1} + C_2 e^{-t/\tau_2} + C_3 e^{-t/\tau_3}$. Three time constants are necessary and sufficient for best possible fits, and indicate three independent relaxation mechanisms that contribute to the force decay (detailed in Refs 2,5). From the fits, all of which have adjusted R-squared values of ≥0.99, we determine time constants associated with each relaxation mechanism, τ_1, τ_2, and τ_3. Time constants plotted for the 0.5:0 (orange) and 0.65:0 (green) DNA blends and the DNA-MT composite (purple) are averaged over all strain rates. As represented by the error bars, all τ_i values show minimal rate dependence. The cessation time Δt_R is chosen to be comparable to the longest measured relaxation time of ~3 s (τ_3 for 0.65:0). The height of each bar represents the mean across 15 trials and error bars correspond to standard error.

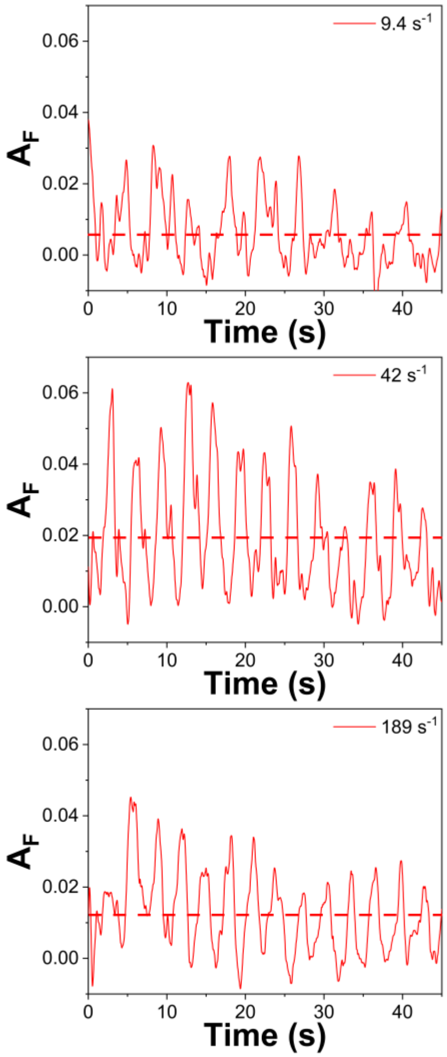

Figure S3. Instantaneous alignment factor values $A_F(t)$ over the course of the entire straining time t that comprises 6-9 periods of strain and cessation. The alignment factor as a function of straining time $A_F(t)$ for the 0.65:0 blend at the closest distance from the strain site ($y = 8$ μm). Each panel corresponds to a different strain rate as listed in the legend. The top and bottom panels are the for the lowest and highest strain rates, and the middle panel is the 'resonant' rate. $A_F(t)$ is computed from the instantaneous 2D image structure function, $D(\mathbf{q}, t, \Delta t)$. For the DDM results presented in the main text, similar to most standard DDM analyses, we average over time to find the image structure function $D(\mathbf{q}, \Delta t)$. Because the videos contain alternating strain and cessation periods, the average A_F values presented in the main text (indicated by the dashed lines in each panel) may be smaller than if we only considered the strain periods. However, despite averaging over periods where the sample is not strained, we still observe statistically significant differences in A_F for different systems, distances from the strain (y), and strain rates ($\dot{\gamma}$) (see Fig 2). Nevertheless, to ensure that time-averaging does not bias our results, we compute the instantaneous (time-dependent) image structure function $D(\mathbf{q}, t, \Delta t)$ to determine the time-varying alignment factor $A_F(t)$. The downside of this approach is increased noise due to not averaging over time, and more labor intensive data analysis. The instantaneous $A_F(t)$ plots show clear signatures of the cyclic strain but the peak values are $\lesssim$3x larger than the average (dashed lines), and follow the same non-monotonic trend with $\dot{\gamma}$.

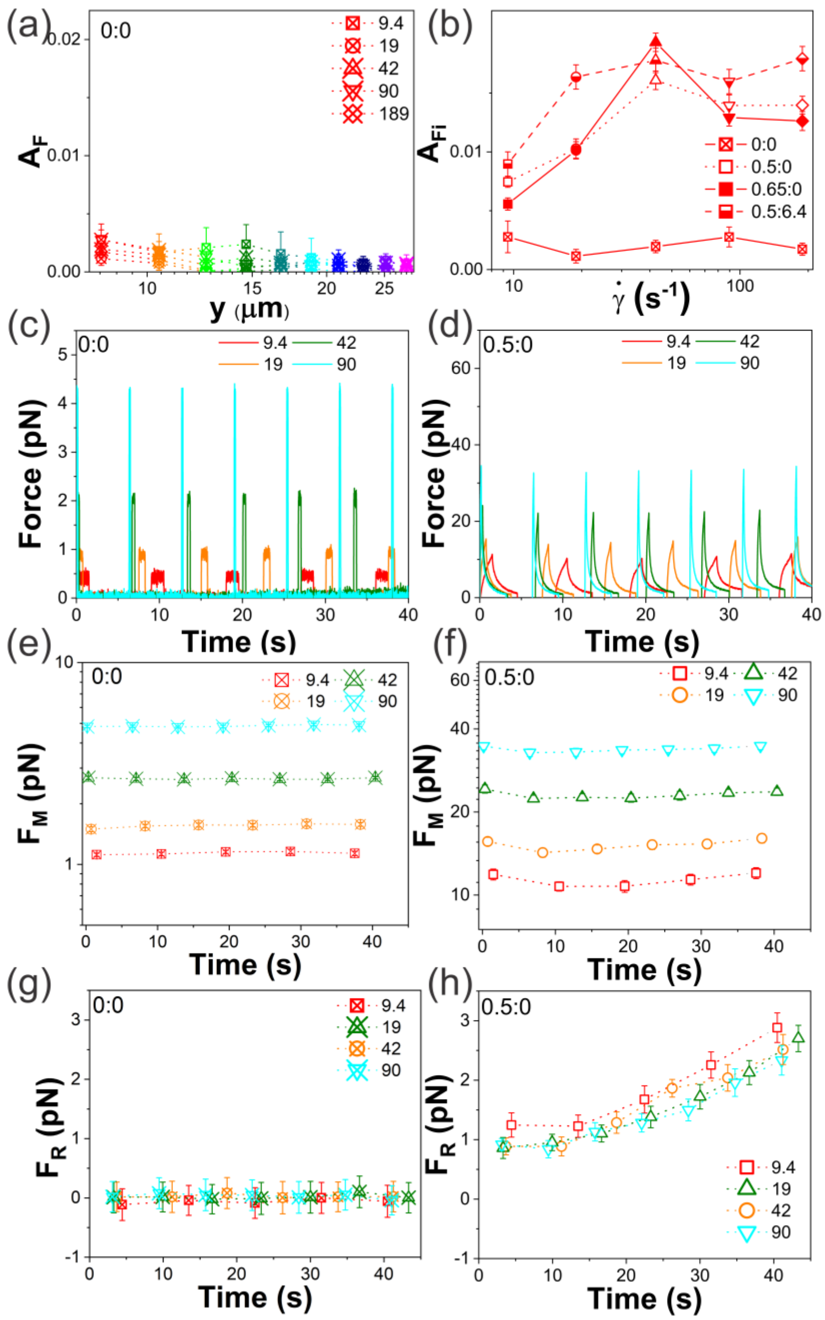

Figure S4. OpTiDDM control measurements in water. (a) $A_F(y)$ for water predictably shows no significant alignment in the direction of strain path for all $\dot{\gamma}$ and y values. **(b)** A_F versus $\dot{\gamma}$ for the closest ROI (y =8 μm) shows that A_F in water is negligibly small and independent of $\dot{\gamma}$ compared to the polymer systems we study here, c_D: c_T = 0.5:0, 0.65:0 and 0.5:6.4 **(c-d)** Sample force traces for cyclic strains in water **(c)** and the 0.5:0 blend **(d)** performed at strain rates listed in the legend in s^{-1}. **(e-h)** Maximum force F_M **(e,f)** and residual force F_R **(g,h)** measured in water (e,g) compared to 0.5:0 (f,h) for strain rates listed in the legends in s^{-1}. Each data point and corresponding error bar shown in (a)-(b) represent the mean and standard error across 5 unique videos. Each data point and corresponding error bar in (e)-(h) represent the mean and standard error across 15 measurements.

Supplementary Information References

1 Robertson, R. M., Laib, S. & Smith, D. E. Diffusion of isolated DNA molecules: dependence on length and topology. *Proc Natl Acad Sci U S A* **103**, 7310-7314, doi:10.1073/pnas.0601903103 (2006).

2 Laib, S., Robertson, R. M. & Smith, D. E. Preparation and characterization of a set of linear DNA molecules for polymer physics and rheology studies. *Macromolecules* **39**, 4115-4119, doi:10.1021/ma0601464 (2006).

3 Peddireddy, K. R., Lee, M., Schroeder, C. M. & Robertson-Anderson, R. M. Viscoelastic properties of ring-linear DNA blends exhibit nonmonotonic dependence on blend composition. *Physical Review Research* **2**, 023213, doi:10.1103/PhysRevResearch.2.023213 (2020).

4 Chapman, C. D., Shanbhag, S., Smith, D. E. & Robertson-Anderson, R. M. Complex effects of molecular topology on diffusion in entangled biopolymer blends. *Soft Matter* **8**, 9177-9182, doi:10.1039/c2sm26279g (2012).

5 Vlassopoulos, D., Pasquino, R. & Snijkers, F. Progress in the Rheology of Cyclic Polymers. *Topological Polymer Chemistry*, 291-316 (2013).

6 Kong, D. J. *et al.* Rheology of Entangled Solutions of Ring-Linear DNA Blends. *Macromolecules* **55**, 1205-1217, doi:10.1021/acs.macromol.1c01672 (2022).

7 Doi, M. & Edwards, S. F. *The Theory of Polymer Dynamics* Vol. 73 (Oxford University Press, 1986).

8 Peddireddy, K. R. *et al.* DNA Conformation Dictates Strength and Flocculation in DNA-Microtubule Composites. *ACS Macro Lett* **10**, 1540-1548, doi:10.1021/acsmacrolett.1c00638 (2021).

9 Wen, Q. & Janmey, P. A. Polymer physics of the cytoskeleton. *Curr Opin Solid State Mater Sci* **15**, 177-182, doi:10.1016/j.cossms.2011.05.002 (2011).

10 Robertson-Anderson, R. M. Optical Tweezers Microrheology: From the Basics to Advanced Techniques and Applications. *ACS Macro Lett* **7**, 968-975, doi:10.1021/acsmacrolett.8b00498 (2018).

11 Rice, A. & Fischer, R. Calibration of Optical Tweezers. *Materials Science Institute* (1997).

12 Squires, T. M. Nonlinear microrheology: bulk stresses versus direct interactions. *Langmuir* **24**, 1147-1159, doi:10.1021/la7023692 (2008).

13 Peddireddy, K. R. *et al.* Unexpected entanglement dynamics in semidilute blends of supercoiled and ring DNA. *Soft Matter* **16**, 152-161, doi:10.1039/c9sm01767d (2020).

14 Ricketts, S. N. *et al.* Varying crosslinking motifs drive the mesoscale mechanics of actin-microtubule composites. *Sci Rep* **9**, 12831, doi:10.1038/s41598-019-49236-4 (2019).

15 Richardson, A. C., Reihani, N. & Oddershede, L. B. in *Optical Trapping and Optical Micromanipulation III.* 632628 (International Society for Optics and Photonics).

16 Chiran, G. & Peixuan, G. Optical tweezer and TIRF microscopy for single molecule manipulation of RNA/DNA nanostructures including their rubbery property and single molecule counting. *Biophysics Reports*, 1-26 (2021).

17 Zieger, V., Betz, T. & Vos, B. E. in *Optical Manipulation and Its Applications.* AM2D. 3 (Optical Society of America).

18 Yang, Z., Piksarv, P., Ferrier, D. E., Gunn-Moore, F. J. & Dholakia, K. Macro-optical trapping for sample confinement in light sheet microscopy. *Biomed Opt Express* **6**, 2778-2785, doi:10.1364/BOE.6.002778 (2015).

19 Wulstein, D. M., Regan, K. E., Garamella, J., McGorty, R. J. & Robertson-Anderson, R. M. Topology-dependent anomalous dynamics of ring and linear DNA are sensitive to cytoskeleton crosslinking. *Sci Adv* **5**, eaay5912, doi:10.1126/sciadv.aay5912 (2019).

20 Varga, Z. & Swan, J. W. Large scale anisotropies in sheared colloidal gels. *Journal of Rheology* **62**, 405-418, doi:10.1122/1.5003364 (2018).

21 Walker, L. M. & Wagner, N. J. SANS Analysis of the Molecular Order in Poly(γ-benzyl l-glutamate)/Deuterated Dimethylformamide (PBLG/d-DMF) under Shear and during Relaxation. *Macromolecules* **29**, 2298-2301, doi:10.1021/ma951127p (1996).

22 Jacob, J. D., He, K., Retterer, S. T., Krishnamoorti, R. & Conrad, J. C. Diffusive dynamics of nanoparticles in ultra-confined media. *Soft Matter* **11**, 7515-7524, doi:10.1039/c5sm01437a (2015).

23 Cho, J. H., Cerbino, R. & Bischofberger, I. Emergence of Multiscale Dynamics in Colloidal Gels. *Phys Rev Lett* **124**, 088005, doi:10.1103/PhysRevLett.124.088005 (2020).

24 Anderson, S. J. *et al.* Filament Rigidity Vies with Mesh Size in Determining Anomalous Diffusion in Cytoskeleton. *Biomacromolecules* **20**, 4380-4388, doi:10.1021/acs.biomac.9b01057 (2019).

25 Anderson, S. J., Garamella, J., Adalbert, S., McGorty, R. J. & Robertson-Anderson, R. M. Subtle changes in crosslinking drive diverse anomalous transport characteristics in actin-microtubule networks. *Soft Matter* **17**, 4375-4385, doi:10.1039/d1sm00093d (2021).

26 He, K. *et al.* Diffusive dynamics of nanoparticles in arrays of nanoposts. *ACS Nano* **7**, 5122-5130, doi:10.1021/nn4007303 (2013).

27 Cipelletti, L., Manley, S., Ball, R. C. & Weitz, D. A. Universal aging features in the restructuring of fractal colloidal gels. *Phys Rev Lett* **84**, 2275-2278, doi:10.1103/PhysRevLett.84.2275 (2000).

28 Lu, P. J. *et al.* Characterizing concentrated, multiply scattering, and actively driven fluorescent systems with confocal differential dynamic microscopy. *Phys Rev Lett* **108**, 218103, doi:10.1103/PhysRevLett.108.218103 (2012).

29 Gao, Y., Kim, J. & Helgeson, M. E. Microdynamics and arrest of coarsening during spinodal decomposition in thermoreversible colloidal gels. *Soft Matter* **11**, 6360-6370, doi:10.1039/c5sm00851d (2015).

30 Cerbino, R. & Cicuta, P. Perspective: Differential dynamic microscopy extracts multi-scale activity in complex fluids and biological systems. *J Chem Phys* **147**, 110901, doi:10.1063/1.5001027 (2017).

31 Cerbino, R., Giavazzi, F. & Helgeson, M. E. Differential dynamic microscopy for the characterization of polymer systems. *Journal of Polymer Science* **60**, 1079-1089, doi:10.1002/pol.20210217 (2021).

32 Chapman, C. D., Lee, K., Henze, D., Smith, D. E. & Robertson-Anderson, R. M. Onset of Non-Continuum Effects in Microrheology of Entangled Polymer Solutions. *Macromolecules* **47**, 1181-1186, doi:10.1021/ma401615m (2014).

33 Iyer, B. V., Lele, A. K. & Juvekar, V. A. Flexible ring polymers in an obstacle environment: Molecular theory of linear viscoelasticity. *Phys Rev E Stat Nonlin Soft Matter Phys* **74**, 021805, doi:10.1103/PhysRevE.74.021805 (2006).
